# General and sports nutrition knowledge among Jordanian adult coaches and athletes: A cross-sectional survey

**DOI:** 10.1371/journal.pone.0258123

**Published:** 2021-11-18

**Authors:** Nour Amin Elsahoryi, Gina Trakman, Ayah Al Kilani

**Affiliations:** 1 Faculty of Pharmacy and Medical Sciences, Department of Nutrition, University of Petra, Amman, Jordan; 2 La Trobe University, School of Allied Health, Bundoora, Australia; Universidade Estadual Paulista Julio de Mesquita Filho - Campus de Bauru, BRAZIL

## Abstract

**Background:**

Nutrition knowledge (NK) is a modifiable determinant of diet intake and can positively influence athletic performance. This study aimed to (1) adapt and translate a validated general and sports NK questionnaire into Arabic (2) assess the NK of Jordanian sportspeople, and (3) evaluate the relationship between NK and various sociodemographic factors.

**Methods:**

The Abridged Nutrition for Sport Knowledge Questionnaire (ANSKQ) was translated into Arabic using forward-backward translation and underwent pilot testing and psychometric validation (internal consistency, test-retest reliability, inter-rater agreement) using a convenience sample of 30 individuals. Following ANSKQ validation, athletes a from 50 sport institutes in Jordan were invited (via email) to complete the Arabic ANSKQ online. Differences in NK based on demographics were analysed using t-test or ANOVA for continuous variables and chi-square tests for categorical variables. The ability of demographic factors to predict NK score-category (poor/good/average/excellent) was assessed using multivariate logistic regression.

**Results:**

The Arabic ANSKQ had excellent internal consistency (Cronbach’s alpha = 0.92), test-retest reliability (Pearson r = 0.926) and inter-rater agreement (Cohen’s k statistic = 0.89). A total of 3636 eligible participants completed the Arabic ANSKQ. Participants were mostly athletes (91.4%), female (68.0%), had normal BMI (50.6%), and played high-intensity sports (59.6%). 88.3% of participants had poor NK (<50%). There were statistically significant differences in NK score based on participant role (athlete vs coach), age, gender, BMI, nationality, smoking, years playing sport, sport frequency, sport intensity, and nutrition training. Multivariate modelling showed participant role, BMI, education level, sport frequency and nutrition training were predictors of NK category.

**Conclusions:**

In conclusion, Jordanian sportspeople have poor NK and may benefit from increased nutrition training.

## Introduction

Following specific diet practices can assist athletes to achieve peak performance [1]. Likewise, when taken using evidence-based protocols, certain ergogenic aids can enhance speed and/or power output and other measures of athletic achievement [2]. Although there are expert and consensus guidelines on athlete’s dietary requirements [1], a high proportion of athletes have sub-optimal energy and carbohydrate intakes [3]. Similarly, the prevalence of (possibly impropriate) dietary supplement use is very high amongst athletes, especially among young athletes [4]. Sport nutrition knowledge appears to have a small but positive association with dietary intake [5–7]. In relation, some researchers have described a negative association between NK and supplement use [8].

Theoretically, providing appropriate nutrition education should increase nutrition awareness regarding food selection, food preparation, meal quality and quantity, and supplement effectiveness and safety. Increased nutrition awareness, can, in turn, support the adoption of more appropriate food and supplement habits [9,10]. Therefore, nutrition education programs are sometimes employed to assist athletes with improving NK [10,11] and support sound dietary intake in athletes [7]. A 2019 systematic literature review found that most nutrition education programs administered to athletes lead to significant improvements in NK [12]. In contrast, findings related to the efficacy of nutrition education interventions in relation to actual diet change are equivocal [8].

In addition to assessing the impact of nutrition education programs undertaken with athletes, many cross-sectional studies have quantified NK of athletes [6,13]. Comparisons across studies are limited due to heterogeneity of tools used to measure NK [6,13]. Overall, however, it appears that athletes across various regions including the North America [14,15], Australia [6,7,13], New Zealand [16], Iran [11], Turkey [17], the UK [18], Ethiopioa [19], and Croatia [13] have significant gaps in their NK. Likewise, there appears to be room for improvement in the knowledge of athletic coaches across America [14,20] and Lebanon [21]. To our knowledge, no studies have been conducted on the NK of athletes or coaches in Jordananian society.

Several researchers have evaluated the association between NK and sociodemographic factors such as general education level, sex, age, as well as the type of sport played and athletic calibre [4,13]. While higher levels of general education have consistently been associated with higher levels of NK, mixed findings have been reported in relation to the association between NK and sex, age, sport played and sporting level [13]; high level analyses of these relationships, including use of regression models, has not been possible in most studies due to relatively low sample sizes. It has been postulated that inconsistencies in NK and reported association between NK and socio-demographic factors may be related to use of diverse questionnaires to measure NK [6,8,12,13]. Ideally, a NK measure should collect information on nutrient function and their relation to health/sporting performance, and also assess procedural skills that are required to translate knowledge into healthier dietary intake [10]. Questionnaires require validation, which is a complex and time-consuming procedure [22]; yet insufficiently validated tools hinder the ability to evaluate the level of NK [10]. Most studies conducted in non-English speaking countries rely on adapted versions of English tools but few report on the translation process and whether re-validation was undertaken. Studies in Arabic-speaking athletes have utilised modified versions of existing tools originally developed in 2003 or self-developed tools [17], and a study in Arabic-speaking coaches used a modified version of a Sports Nutrition Knowledge Questionnaire, developed in 2005 [21]. Overall, the NK questionnaires used in Arabic athletes and coaches have either been poorly validated or are not based on current sports nutrition guidelines.

Considering the necessity of employing validated and culturally relevant questionnaires to assess NK, and the current lack of both a valid and up-to-date Arabic sports NK questionnaire, as well as the scarcity of data on Jordanian athletes and coaches, the aims of the current study are to: (1) adapt and translate a validated general and sports NK questionnaire into Arabic, (2) assess the NK of Jordanian sportspeople, and (3) evaluate the relationship between NK and various sociodemographic factors.

## Materials and methods

### Study design

This cross-sectional study had two parts: (1) Development of Arabic General and Sports Nutrition Knowledge Questionnaire and (2) Evaluation Jordanian athletes’ and coaches’ NK and assessment of the association between NK and demographic characteristics.

### Development of Arabic General and Sports Nutrition Knowledge Questionnaire

#### English ANSKQ

The ANSKQ has been previously validated used Rasch Analyses in 181 members of one non-elite Australian football and netball club [23]. The questionnaire was found to have two-dimensional sections (general nutrition knowledge, and sport nutrition knowledge) which both fit the Rasch model. Test-retest reliability was confirmed (r = 0.7 to 0.8) and construct validity was demonstrated using known-group comparisons (p-value< 0.001) [23]. In 2019 the ANSKQ underwent minor modifications and updates [24]. The modified ANSKQ includes a demographic section (regarding sex, gender, age, education level, sporting characteristics and information source) as well as 35 knowledge items on general (n = 11) and sports (n = 24) NK. Response categories include agree/disagree/not sure or high/low/not sure or enough/not enough/not sure or yes/no/not [24]. The English ANSKQ is available as supporting material.

#### Translation and assessment of face, content validity of the ANSKQ

The modified ANSKQ was translated into Arabic using the forward-backward translation method [25]. First, the questionnaire was translated from English to Arabic by a bilingual researcher (Arabic, English). A second bilingual researcher retranslated the questionnaire back from Arabic to English. The two English versions (original, translated-back translated) were then compared to ensure the correct meaning of the items.

#### Further psychometric validation of the Arabic ANSKQ

The Arabic ANSKQ was tested on a pilot sample of 30 adults. The pilot study took place between August 2020 and September 2020. Participants were contacted 10 working days after first completing the tool and sent the questionnaire again. Construct validity was evaluated by estimating the association between the scale items (Correlation matrices). The reliability was assessed by Internal consistency (Cronbach’s alpha), test-retest reliability (Pearson’s r), and inter-rater reliability (Cohen’s k statistic).

### Evaluation Jordanian athletes’ and coaches’ nutrition knowledge and assessment of the association between nutrition knowledge and demographic characteristics

#### Participants and setting

**Fig 1:**
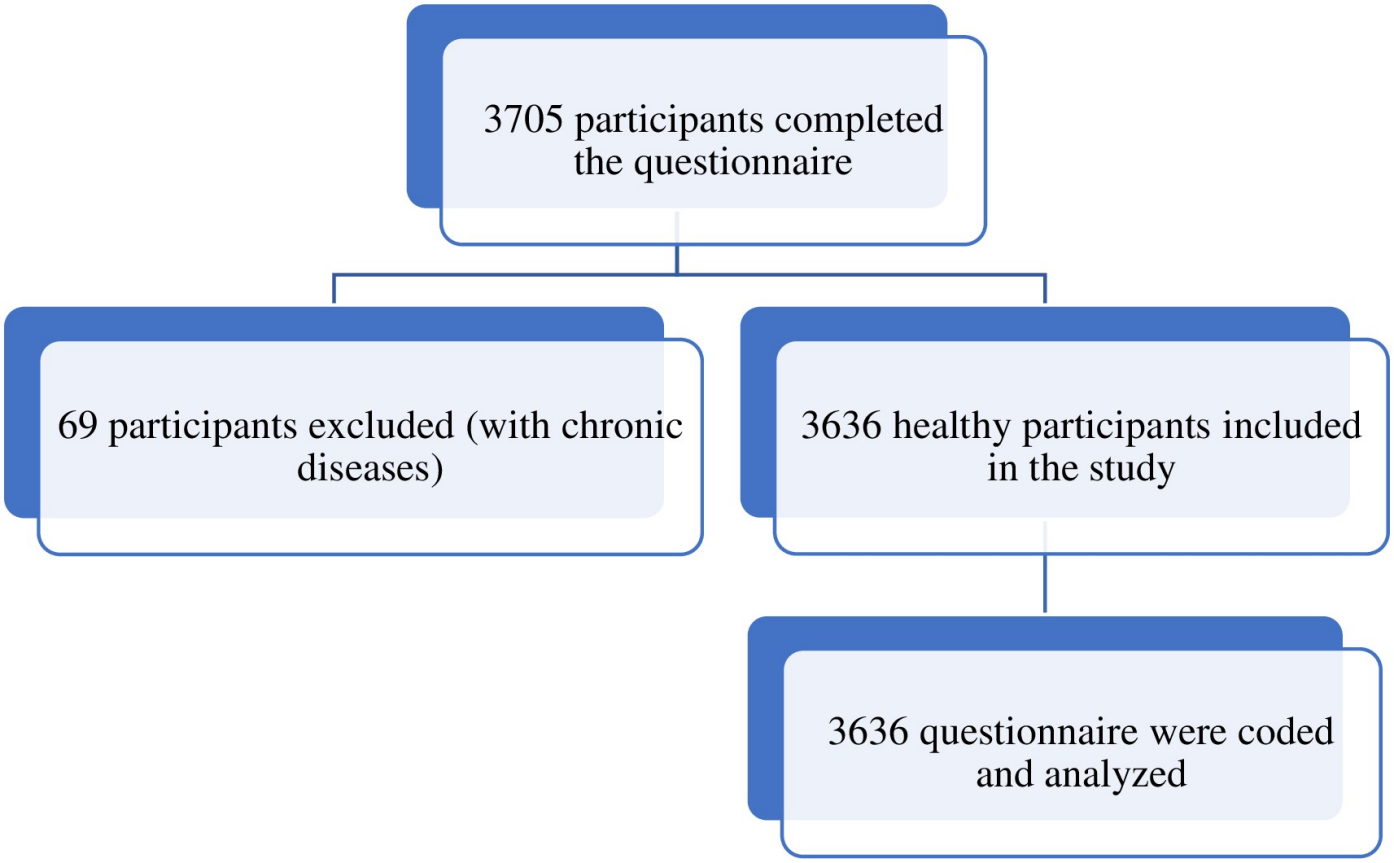
The recruitment of the athletes and coaches for nutrition knowledge assessment.

Recruitment for assessment of athletes and coaches NK took place between October 2020 and December 2020. Due to the lack of a population sampling framework, and requirements for large sample size to properly assess the association between demographic factors and NK, the respondents were selected using convenience sampling (non-probability sampling method) [26]. The inclusion criteria were healthy athletes, aged between 18 and 60 years old as shown (Fig 1). Exclusion criteria were individuals with chronic disease, outside the desired age range, and individuals who were not athletes or coaches. Athletes in this study have been defined as persons who exercise to improve their sporting performance and spend time playing a particular sport as part of an organised competition [27].

An invitation to participate in the study was sent to 50 sports centres in Jordan via electronic invitation using social media platforms (Facebook, WhatsApp, Twitter, Snapchat, and Instagram). The sport institutes included Universities, Colleges, national and local sport centres, sport centres in the Al Hussein Youth City (Amman) and Al Hassan Youth City (Irbid). The centres that were invited included all sport types. Athletes and coaches were informed that participation in the study was voluntary, without any type of incentives (monetary or non-monetary) offered. The translated ANSKQ was self-administered using Qualtrics. A participant information statement and consent form were included on the first page of the questionnaire, with participants asked to confirm that they were willing to participate (by completing the ANSKQ) before being directed to demographic questions and knowledge items. The participants were reported on the first page that they are free to leave the study at any time by exiting the online questionnaire.

### Data handling and statistical analysis

Responses to the ANSKQ were directly exported to Statistical Package for Social Sciences (SPSS Inc., Chicago, IL, US), version 26.0. All the information was preserved in a secure Google Drive to which only the lead investigator had access.

The Kolmogorov-Smirnov and Shapiro-Wilk test was used to determine the normality of the numerical data, where a value of P> 0.05 indicates normally distributed data. All our data was normally distributed and therefore reported as mean and standard deviation (SD), with parametric tests applied.

Participant’s sociodemographic data were reported as n (%) using descriptive statistics for continuous variables and frequency analyses for categorical variables. Preferred information sources underwent frequency analyses. Total NK scores were converted to percentages and then classified based on [23] as follows: poor (0 and 49%), average (50 and 65%), good (66 and 75%), and excellent (above 75%). The classifications were derived based on results of the validation of the NSKQ, whereby mean scores of subsections ranged from 46–63% for those without nutrition education and 54–78% for those with nutrition education.

#### Univariate analysis

Two-tailed t-test, One- way analysis of variance (ANOVA) and chi-square tests were applied to assess differences in mean NK score based on demographic characteristics. Cross-tabulation (Chi Square (χ2) used to assess whether there where statistical differences in the NK categories based on demographic characteristics of the participants. ANOVA analysis was used with post hoc Student Newman Keuls to determine whether there were any statistically significant differences in socio-demographic characteristics with the mean total NK score obtained on the ANSKQ. The effect size (Cohen’s D) for independent t-test and Partial Eta Squared (η2p) for ANOVA test) was calculated to provide a measure of the practical significance of the univariate analysis.

#### Multivariate logistic regression analysis

Multivariate logistic regression models were used to identify potential independent determinants of the knowledge level among the athletes and coaches in Jordan. All variables determined to be significant at p-value≤ 0.05 in the univariate analyses were included in the multivariate models. For each participant characteristic, Odds Ratio (OR) estimated the likelihood of having average total knowledge, good total knowledge, or excellent total knowledge (relative to having poor NK). All statistical significance was established at a confidence interval of 95% and p-value≤ 0.05.

### Ethical approval

The Research Ethics Committee of the Faculty of Pharmacy and Medical Sciences, Petra Internal Review Board (IRB), University of Petra, Amman, Jordan, gave its approval to this study (Grant number: Q1/9/2020). In this investigation, all ethical principles of the Helsinki statement and its revisions from 1964 were observed.

## Results

### Development of Arabic General and Sports Nutrition Knowledge Questionnaire

#### Translation and assessment of face, content validity of the ANSKQ

The accuracy of the Arabic ANSKQ was confirmed by a panel of five experts of nutrition lecturers. Based on the nutritionist feedback, two questions, regarding alcohol intake, were omitted from the general knowledge section because they are not consistent with the religion and the food habits of the Jordanian culture, and they could violate the privacy of the participants. After these edits, the panel of nutrition experts confirmed face, content validity by concluding that the questionnaire was: clear and easy to understand, covered most points in the subject, could be used in the future, and would not violate participants’ privacy. The Arabic ANSKQ is available as supporting material.

#### Further psychometric validation of the Arabic ANSKQ

The correlation coefficients (r^2^) ranged between 0.55 and 0.896, with a mean value of 0.72, which reflects moderate construct validity. The questionnaire was reliable regarding overall internal consistency (Cronbach’s alpha = 0.92) and test-retest reliability (Pearson (r^2^) = 0.926) and the inter-rater agreement (Cohen’s k statistic = 0.89). The coefficient alpha result reflects excellent internal consistency. The results of the stability coefficient indicated strong test-retest reliability, reflecting that measurement error of the questionnaire is less likely to be attributable to changes in NK.

### Evaluation Jordanian athletes’ and coaches’ nutrition knowledge and assessment of the association between nutrition knowledge and demographic characteristics

#### Participant characteristics

Participant characteristics and mean NK scores are summarised in Table 1. As shown in Fig 1, a total of 3705 participants completed the survey, 69 participants were excluded from the study because they didn’t meet the inclusion criteria and 3636 participants included in the analysis. Most participants were athletes (91.4%). Participants were evenly disturbed amongst four age categories. More than half of the participants were females (68%) and had a normal BMI (59.6%). The dominant nationality was Jordanian (64%) and the education level of more than one-third of the participants was diploma or bachelors (80.8%). More than half students involved in medical courses (63.8%) and more than half of the participants were non-smokers (62.6%). Regarding the sport behaviour, the largest percentage of the participants are playing high intensity sport (47.9%), 1–2 times per week (47.6%). Almost half of the participants consumed dietary supplements (52.2%) but 80.5% did not consume doping agents. Most participants (82.4%) had not received training courses regarding sports nutrition and around two-thirds of the participants had not received nutrition consultation (75.2%).

**Table 1:** Demographic and lifestyle characteristics of the study participants and univariate analysis of influence of factors on mean total nutrition knowledge score obtained on the Abridged Nutrition Knowledge Questionnaire (ANSKQ) (n = 3636).

Characteristics	Categories	All participants n (%)	*Total mean correct score ± SD	F (df)	p-value	**Effect size
Participant role	Coach Athlete	313 (8.6) 3323 (91.4)	51.92±26.06 35.19±12.76	578.71 (3634)	≤ 0.001	0.82
Age	18–25 years 26–35 years 36–50 years	1344 (37) 1300 (35.8) 992 (27.3)	35.16±14.07 ^a^ 37.88±16.11 ^b^ 36.97±15.05 ^b^	11.1 (2)	≤ 0.001	0.01
Gender	Male Female	1165 (32) 2471 (68)	38.17±14.56 35.90±16.18	10.73 (3)	≤ 0.001	0.16
BMI (kg/m^2^)	<18.5 18.5–24.9 25.0–29.9 ≥30.0	74 (2) 2168 (59.6) 1030 (28.3) 364 (10)	42.87±17.30 ^a^ 36.86±15.28 ^b^ 36.90±14.64 ^b^ 33.22±14.56 ^c^	10.73 (3)	≤ 0.001	0.02
Nationality	Jordanian Non-Jordanian	2334 (64) 1302 (35.8)	37.33±15.25 36.24±15.05	0.05 (3634)	0.04	0.07
Education level	High-school or less Diploma or bachelors Postgraduate	475 (13.1) 2937 (80.8) 224 (6.2)	34.37±13.73 ^a^ 36.68±15.20 ^b^ 40.73±16.25 ^c^	13.66 (2)	≤ 0.001	0.02
Study course	Medical Non-medical	2320 (63.8) 903 (24.8)	35.99±13.98 36.99±15.74	6.67 (3634)	0.06	0.07
Smoking	Yes No	1361 (37.4) 2275 (62.6)	37.31±14.94 36.22±15.23	0.32 (3634)	0.04	0.07
Sport years	≤ 1 year 2–3 years 4–9 years >10 years	1748 (48.1) 1170 (32.2) 342 (9.4) 376 (10.3)	34.91±14.45 ^a^ 37.50±14.47 ^b^ 39.52±17.12 ^b^ 39.29±17.22 ^b^	17.06 (3)	≤ 0.001	0.02
Sport type	High intensity Medium intensity Low intensity	1741 (47.9) 884 (24.3) 1011 (27.8)	35.42±14.14 ^a^ 36.99±14.02 ^b^ 38.40±17.37 ^c^	12.85 (2)	≤ 0.001	0.02
Sport frequency/week	1–2 time/week 3–5 time/week >5 times/week	1730 (47.6) 1484 (40.8) 422 (11.6)	35.40±14.07 ^a^ 37.20±15.46 ^b^ 39.65±17.50 ^c^	15.26 (2)	≤ 0.001	0.02
DS intake	Yes No	1898 (52.2) 1738 (47.8)	36.29±14.70 37.0±15.59	3.06 (3634)	0.16	0.05
Doping agents	Yes No	708 (19.5) 2928 (80.5)	36.40±14.72 36.68±15.23	0.87 (3634)	0.66	0.02
Nutrition training	Yes No	640 (17.4) 2996 (82.4)	45.82±22.38 34.66±12.21	395.64 (3634)	≤ 0.001	0.62
Nutrition consultation	Yes No	903 (24.8) 2733 (75.2)	36.19±15.35 36.77±15.06	0.29 (3634)	0.31	0.04

Categorical variables represented as n (%). Significance set at p-value≤ 0.05. Relationship for two categories run by using independent t test. For more than two categories, one-way ANOVA was used.

*Normal distributed approved by Kolmogorov-Smirnov and Shapiro-Wilk test. Doping agents: Performance-enhancing. This classification was based on [28]. DS: Dietary supplements. BMI: Body Mass Index. F: The test values (chi-squared for two groups and ANOVA for three groups and more). df: Degree of freedom.

**Cohen’s D (d) for independent t-test (Cohen’s d) and Partial Eta Squared (η2p) for ANOVA test. One way ANOVA was used to compare between intervention groups with post hoc Student Newman Keuls tests (age, BMI, education level, sport year, sport type, and sport frequency). Heterogeneous subsets are indicated using different superscripted letters.

#### Nutrition knowledge and association between demographic factors and nutrition knowledge

The total mean NK score across participant characteristics ranged from 33.22% (participants with BMI >30kg/m2) to 51.92% (coaches). There was a significant difference in total mean NK based on several participant characteristics. Coaches scored higher than athletes (51.92% vs 35.19%, t (3643) = 578.71, p-value≤ 0.001, Cohen’s d = 0.82; males scored higher than females (38.17% vs 35.9%, t (3) = 10.73, p-value≤ 0.001, Cohen’s d = 0.85; Jordanians scored higher than non-Jordanians (37.33% vs 36.42%, t (3643) = 0.05, p-value = 0.04, Cohen’s d = 0.07; smokers scores higher than non-smokers (37.31% vs 36.22%, t (3643) = 0.32, p-value = 0.04, Cohen’s d = 0.07; and individuals with nutrition training scored higher than individuals without nutrition training (45.82% vs 34.66%, a t (3643) = 395.64, p-value≤ 0.001, Cohen’s d = 0.62. There were also statistically significant differences based on age categories (F(2) = 11.1, p-value≤ 0.001, η2p = 0.01), BMI categories (F(3) = 10.73, p-value≤ 0.001, η2p = 0.02), duration playing sport in years (F(3) = 17.06, p-value≤ 0.001, η2p = 0.02), sport type (F(2) = 12.85, p-value≤ 0.001, η2p = 0.02) and frequency playing sport per week (f F(2) = 15.26, p-value≤ 0.001, η2p = 0.02). The mean knowledge score was higher amongst participants aged from 26 to 35 years old, underweight participants, participants who had played sports for four years or more, and participants who played sport more than five times per week (Table 1). There was a large effect size (≥ 0.8) for participant’s role (athletes/couches) and a medium to large effect size for having undertaken nutrition training. Effect size for all other variables (gender, participants age, BMI, nationality, education level, study year, sport type and sport frequency/week) was small.

#### General and sport nutrition knowledge classification

Based on the cut-off of the general and sports nutrition knowledge score described by Trakman et al. [22], the results indicated that 88.3% of the participants had poor knowledge (< 50%) and 3.2% had excellent NK (> 75%) (Table 2).

**Table 2:** The cut-off of the knowledge mean percentage and the distribution of the study participant among the knowledge categories.

Knowledge category	Cut-off of the total knowledge mean percentage	Total Knowledge mean percentage, n (%)
Poor knowledge	Between 0 and 49%.	3210 (88.3)
Average knowledge	Between 50 and 65%.	273 (7.5)
Good knowledge	Between 66 and 75%.	37 (1.0)
Excellent knowledge	Above 75%.	116 (3.2)

The knowledge categorized based on the total mean of all questions.

#### Factors associated with nutrition knowledge categories

*Univariate analysis of the nutrition knowledge based on participant’s characteristics*. Table 3 shows the relationship between participant sociodemographic and lifestyle characteristics and the knowledge categories according to the univariate analysis. (χ^2^) analysis results indicated that the participant role (p-value≤ 0.001), age (p-value = 0.001), gender (p-value = 0.01), BMI (p-value = 0.001), education level (p-value = 0.01), sport year (p-value≤ 0.001), sport type (p-value≤ 0.001), sport frequency per week (p-value≤ 0.001) and attending nutrition training course (p-value≤ 0.001) were significantly different among NK score categories (p-values≤ 0.001). There was no significant relationship between NK level and the following participant’s characteristics: nationality (p-value = 0.31), study course (p-value = 0.15), smoking (p-value = 0.88), DS intake (p-value = 0.41), doping agents’ intake (p-value = 0.82) and availability of nutrition consultation (p-value = 0.18).

**Table 3:** Univariate analysis of relationship between participant sociodemographic, lifestyle characteristics and knowledge categories achieved on the Abridged Nutrition Knowledge Questionnaire (ANSKQ).

Characteristics	Categories	Poor knowledge n = 3210	Average knowledge n = 273	Good knowledge n-37	Excellent knowledge n = 116	Χ^2^ (df)	p-value
Participant role	Coach Athlete	190 (60.7) 3020 (90.9)	27 (8.6) 246 (7.4)	15 (4.8) 22 (0.7)	81 (25.9) 35 (1.1)	630 (3)	≤ 0.001
Age	18–25 years 26–35 years ≥36 years	1221 (90.8) 1123 (86.4) 866 (87.3)	85 (6.3) 102 (7.8) 86 (8.7)	8 (0.6) 22 (1.7) 7 (0.7)	30 (2.2) 53 (4.1) 33 (3.3)	22.38 (6)	0.001
Gender	Male Female	2210 (89.4) 1000 (85.8)	172 (7) 101 (8.7)	24 (1) 13 (1.1)	65 (2.6) 51 (4.4)	11.98 (3)	0.01
BMI (kg/m^2^)	<18.5 18.5–24.9 25.0–29.9 ≥30.0	57 (77) 1897 (87.5) 914 (88.7) 342 (94)	9 (12.2) 179 (8.3) 71 (6.9) 14 (3.8)	3 (4.1) 19 (0.9) 1 (0.3) 37 (1)	5 (6.80 73 (3.4) 31 (3) 7 (1.9)	28.72 (9)	0.001
Nationality	Jordanian Non-Jordanian	2070 (88.9) 1134 (87.1)	166 (7.1) 107 (8.2)	20 (0.9) 17 (1.3)	72 (3.1) 44 (3.4)	3.57 (3)	0.31
Education level	High-school or less Diploma or bachelors Postgraduate	435 (91.6) 2592 (88.3) 183 (81.7)	28 (5.9) 219 (7.5) 26 (11.6)	4 (0.8) 28 (1) 5 (2.2)	8 (1.7) 98 (3.3) 10 (4.5)	16.85 (6)	0.01
Study course	Medical Non-medical	1176 (89.4) 2034 (87.7)	98 (7.4) 175 (7.5)	10 (0.8) 27 (1.2)	32 (2.4) 84 (3.6)	5.34 (3)	0.15
Smoking	Yes No	1195 (87.8) 2015 (88.6)	105 (7.7) 168 (7.4)	14 (1) 23 (1)	69 (3) 47 (3.5)	0.66 (3)	0.88
Sport years	≤ 1 year 2–3 years 6–9 years >10 years	1583 (90.6) 1017 (86.9) 292 (85.4) 318 (84.6)	108 (6.2) 109 (9.3) 26 (7.6) 30 (8)	16 (0.9) 12 (1) 2 (0.6) 7 (1.9)	41 (2.3) 32 (2.7) 22 (6.4) 21 (5.6)	37.65 (9)	≤ 0.001
Sport type	High intensity Medium intensity Low intensity	1575 (90.5) 776 (87.8) 859 (85)	113 (6.5) 78 (8.8) 82 (8.1)	19 (1.1) 8 (0.9) 10 (1)	34 (2) 22 (2.5) 60 (5.9)	40.95 (6)	≤ 0.001
Sport frequency/week	1–2 time/week 3–5 time/week >5 times/week	157 (91) 1291 (87) 345 (81.8)	102 (5.9) 126 (8.5) 45 (10.7)	16 (0.9) 12 (0.8) 9 (2.1)	38 (2.2) 55 (3.7) 23 (5.5)	36.53 (6)	≤ 0.001
DS intake	Yes No	1683 (88.7) 1527 (87.9)	145 (7.6) 128 (7.4)	18 (0.9) 19 (1.1)	52 (2.7) 64 (3.7)	2.87 (3)	0.41
Doping agents Intake	Yes No	629 (88.8) 2581 (88.1)	52 (7.3) 221 (7.5)	5 (0.7) 32 (1.1)	22 (3.1) 94 (3.2)	0.92 (3)	0.82
Nutrition training	Yes No	469 (73.3) 2741 (91.5)	29 (4.5) 244 (8.1)	33 (5.2) 4 (8.1)	109 (17) 7 (0.2)	7.24 (3)	≤ 0.001
Nutrition consultation	Yes No	1210 (89) 2000 (87.8)	97 (7.1) 176 (7.7)	17 (1.3) 20 (0.9)	34 (2.5) 82 (3.6)	7.92 (3)	0.18

Categorical variables represented as n (%). χ2: Chi squared. Significance set at p-value≤ 0.05. Total % per row. DS: Dietary supplements. BMI: Body Mass Index. df: Degree of freedom.

*Multivariate logistic regression analysis of nutrition knowledge based on participant’s characteristics*. Table 4 shows the results of the multivariate logistic regression model, which calculated odds ratios of achieving average, good, and excellent total NK, relative to poor total NK. Model fitting information results were significant (χ^2^) = 756.38, df = 51, p-value≤ 0.001.

**Table 4:** Multivariate logistic regression of the ability of participants sociodemographic and lifestyle characteristics to predict knowledge categories obtained on the Abridged Nutrition Knowledge Questionnaire (ANSKQ).

Variable	Categories	Average knowledge (0–49)		Good knowledge (50–65)		Excellent knowledge (66–75)	
		B	Exp(B) (95% CI)	B	Exp(B) (95% CI)	B	Exp(B) (95% CI)
Participant role	Coach Athlete	0.40 0^b^	1.49 (0.96, 2.32) -	2.24 0^b^	9.35 (4.13, 21.16)*	3.56 0^b^	35.05 (19.82, 61.99)*
Age	18–25 years 26–35 years ≥ 36 years	-0.19 -0.17 0^b^	0.82 (0.59, 1.16) 0.85 (0.62, 1.15) -	0.01 0.89 0^b^	1.01 (0.32, 3.20) 1.02 2.44 (0.78, 6.09)	-0.05 0.12 0^b^	0.95 (0.45, 1.99) 1.12 (0.60, 2.09)
Gender	Female Male	-0.07 0^b^	0.94 (0.71, 1.24) -	-0.03 0^b^	0.97 (0.43, 2.18) -	0.01 0^b^	1.03 (0.57, 1.82)
BMI (kg/m^2^)	<18.5 18.5–24.9 25.0–29.9 ≥ 30.0	1.07 0.72 0.61 0^b^	2.92 (1.17, 7.32)* 2.05 (1.15, 3.64)* 1.84 (1.02, 3.31)* -	0.80 0.66 1.21 0^b^	2.23 (0.18, 28.35) 1.94 (0.24, 15.88) 3.35 (0.42, 27.02) -	-0.98 -0.09 -0.32 0^b^	0.38 (0.07, 2.03) 0.92 (0.32, 2.63) 0.73 (0.25, 2.13)
Education level	High-school or less Diploma or bachelors Postgraduate	-0.65 -0.39 0^b^	0.52 (0.29, 0.93)* 0.68 (0.43, 1.06) -	-0.30 -0.45 0^b^	0.74 (0.17, 3.32) 0.64 (0.21, 1.97) -	0.35 0.56 0^b^	1.42 (0.39, 5.18) 1.76 (0.67, 4.64) -
Sport years	≤ 1 year 2–3 years 6–9 years >10 years	0.13 0.37 0.03 0^b^	1.14 (0.70, 1.85) 1.45 (0.92, 2.28) 1.03 (0.59, 1.81) -	-0.05 0.02 -0.84 0^b^	0.95 (0.27, 3.39) 1.02 (0.32, 3.26) 0.43 (0.08, 2.46) -	0.32 0.20 0.69 0^b^	1.38 (0.54, 3.49) 1.23 (0.51, 2.95) 1.99 (0.76, 5.19) -
Sport type	High intensity Medium intensity Low intensity	-0.04 0.13 0^b^	0.96 (0.68, 1.35) 1.14 (0.81, 1.61) -	0.61 0.25 0^b^	1.83 (0.67, 5.01) 1.28 (0.44, 3.73) -	-0.52 -0.46 0^b^	0.60 (0.30, 1.19) 0.63 (0.31, 1.30) -
Sport frequency/week	1–2 time/week 3–5 time/week > 5 times/week	-0.55 -0.30 0^b^	0.58 (0.38, 0.87)* 0.74 (0.50, 1.08) -	-0.83 -0.76 0^b^	0.44 (0.14, 1.36) 0.47 (0.16, 1.35) -	-0.35 0.08 0^b^	0.71 (0.29, 1.70) 1.09 (0.50, 2.39) -
Nutrition training Courses	No Yes	0.36 0^b^	1.44 (0.96, 2.14) -	-3.83 0	0.02 (0.01, 0.06)* 0	-4.66 0^b^	0.01 (0.004, 0.02)*

**Notes**: The poor category is reference category; This parameter is set to zero because it is redundant; CI = Confidence interval; OR = Odds ratio.

*p-value≤ 0.05.

B: The estimated multinomial logistic regression coefficients for the models. BMI: Body Mass Index.

Compared to athletes, coaches were 9.35 times more likely to have good rather than poor knowledge, and 33.05 times more likely to have excellent rather than poor knowledge.

Compared to participants who were obese, participants who were underweight, normal weight and overweight were 2.92 times, 2.05 times, and 1.841 times more likely to have average (rather than poor) NK, respectively.

Compared to participants who had a post-graduate degree, participants who had high-school education were 0.52 times less likely to have average (rather than poor) knowledge.

Compared to participants who trained for more than five time weekly, participants who trained 1–2 times weekly were 0.58 times less likely to have average (rather than poor) knowledge.

Compared to participants who had attended nutrition courses, those who had not attended nutrition training courses were 3.83 times less likely to have good (rather than poor) knowledge. Compared to participants who had attended nutrition courses, participants who had not attended nutrition training courses were also 4.64 times more likely to have excellent (rather than poor) NK.

#### Nutrition- related information sources

**Fig 2:**
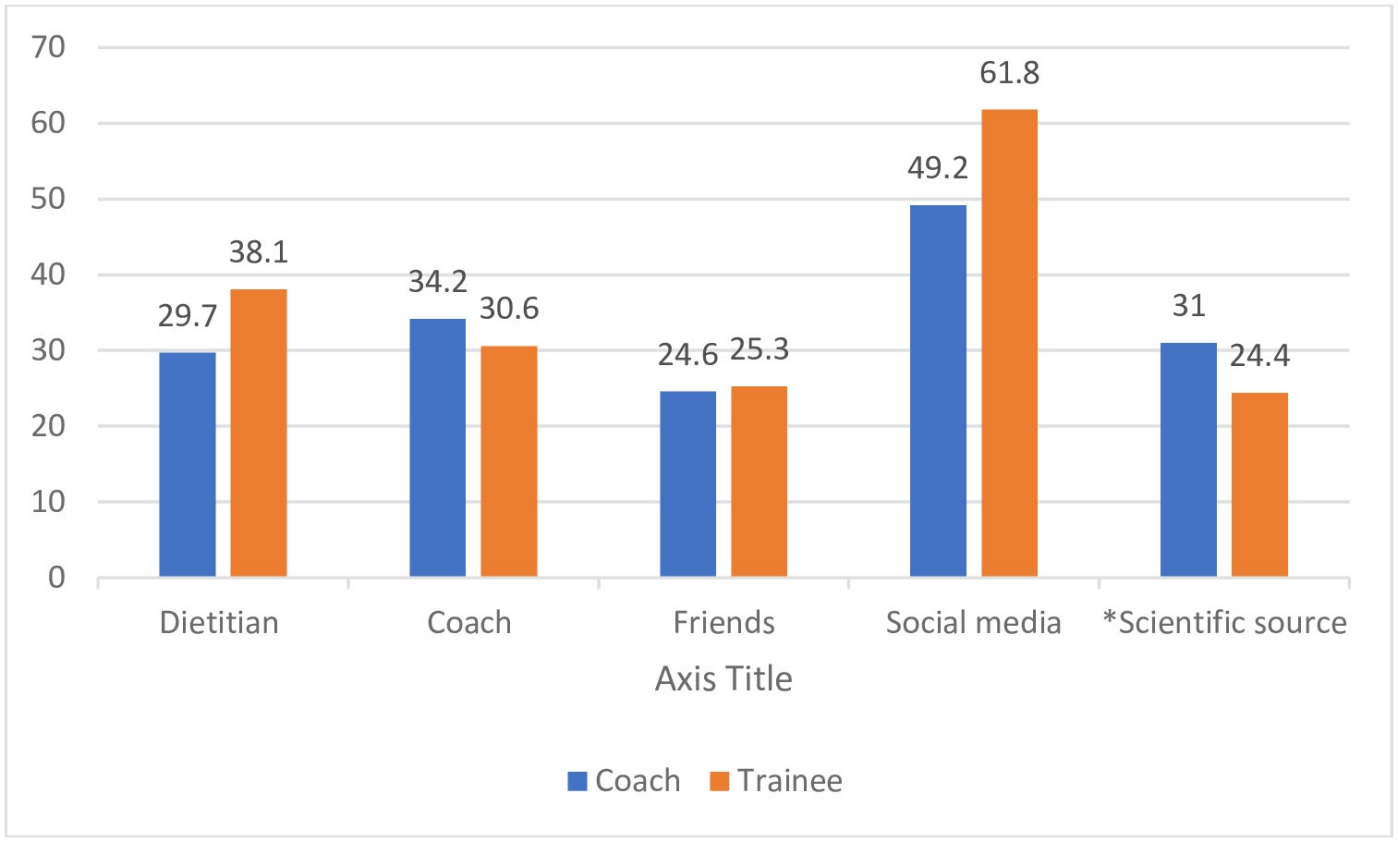
The percentage of the Jordanian athletes who say they use information sources for nutrition-related issues. *Scientific information includes the formal scientific webpages, published research, published books, and documentation).

All participants were asked about their main sources of nutrition information. There was no order required in this question and multiple responses were allowed. Coaches predominately used social media (49.2%), with a roughly equal preference for other coaches (31.0%), scientific sources (31.0%) and dietitians (29.7%); only 24.6% chose friends as a source of information Among the athletes, about two -thirds (61.8%) used social media, followed by dietitian (38.1%), coaches (30.6%), friends (25.3%) and scientific sources (24.4%) (Fig 2).

## Discussion

To our knowledge, this is the first study to assess the NK of Jordanian athletes and coaches. The key findings are: (1) Most participants had poor NK but coaches had better knowledge than athletes. (2) A multivariate logistic regression model revealed that BMI, nutrition training, general education level, and frequency of training for sport significantly affected odds of achieving average, good, and excellent total NK, relative to poor total NK. (3) social media was a primary source of information for most participants.

The overall mean NK score of athletes (35.2%) in our study was lower than has previously been reported in other studies that have used the ANSKQ (or its longer version, the NSKQ) [18,23,29–31]. Nutrition knowledge scores in international studies ranged from 40.2% in Irish Gaelic Football players [32] to 56.7% English squash player (29). No studies in Jordanian athletes exist for direct comparison. The ANSKQ has also not been used in any studies evaluating coaches NK; coaches NK scores in the present study (51.92%) appear comparable to scores obtained by various athletes in international studies where the NSKQ has been used.

The relatively poor NK scores may be explained by participants preferred information sources. About 62% of athletes and 50% of coaches reported using social media as an information source. Previous research has found that the internet is also the primary information source for cross-fit trainers, high school coaches, and Australian athletes [24]. Both the internet and social media are known to contain misinformation, especially when content is designed for the promotion and sale of supplements. Interestingly, while a significant portion of respondents had a high-level of general education (81% diploma or bachelor’s degree, 6% postgraduate qualifications), only 31% of coaches and 24% of athletes relied on reputable sources (i.e. scientific sources) for nutrition information.

Ostensibly, a further, reasonable explanation for relatively poor NK scores is that participants may lack of access to nutrition professionals, with only 25% of participants reporting they had nutrition consultations. However, there were not actually any significant differences in NK between those who had and had not had nutrition consultations. Similar findings were reported by Trakman et al. [13,23,24,33], who found no significant differences between athletes who had/had not been advised by a dietitian in the past [24]. Trakman et al. [24] proposed that nutrition consultations may (paradoxically) not lead to better nutrition knowledge because sports nutrition is complex, and requires professionals to undergo specialised training and education, which not all professionals providing advice to athletes will have done. Further, nutrition education may focus on what foods to eat/meal planning, rather than the theory of sports nutrition.

We found a difference in NK based on several demographic factors. For age, gender, nationality, education level, smoking, sport type and sport frequency, the differences were numerically very small (1–3%), with small effect sizes and thus unlikely to have practical significance. The differences for participant type (athlete versus coach), previous nutrition training, and BMI had a larger magnitude (6–10%), with the former two having medium to large effect sizes, indicating a more substantial difference. Participant type, BMI, nutrition training, along with frequency of training and level of education were the five factors that were also significant in the multivariate regression model; in line with effect size results, odds ratio for participant type and BMI were substantial, but these were small for other variables. Existing studies have reported mixed findings in relation to the association between participant’s age, BMI, gender, sport played and sporting level and general/sports nutrition knowledge. In contrast being a coach (rather than athlete), higher general level of education and nutrition training appear to also be consistently associated with better NK [4,5,9,10,14,18,32–37] in the broader literature. Of note, many of these existing studies used relatively small sample sizes and did not report effect sizes or undertake predictive modelling, as we have done.

It is plausible that athletes with a lower BMI have a higher NK because they are interested in nutrition and thus seek out information on healthy eating patterns and nutrition for sports performance. Valmórbida et al. 2017 found that cardiology patients in Brazil with a higher BMI had lower NK. However, as noted, several studies undertaken on athletes have not reported this relationship. There were significant differences in coaches versus athletes in our univariate, but not multivariate analyses. This may indicate that co-variates associated with being a coach (i.e. level of education, general nutrition education) may explain their apparent higher levels of NK. Indeed, coaches do often have access to nutrition training or education courses, which have positive impacts on their nutrition knowledge [4]. Further, many coaches provide nutrition information to their athletes [14], and thus may be more likely to seek out education on sports nutrition. Level of general education may be associated with high NK due to better general comprehension of question items [10], or increased likelihood of having sought of nutrition education. Likewise, undertaking nutritional training is likely to support awareness and knowledge of their dietary habits and patterns; a systematic literature view confirmed that nutrition education programmes increase NK [12]. More frequent training may indicate high athletic calibre, which has been associated with increased NK (in some [35] but not all [29] due to improved access to resources and increased interest in nutrition.

Taken together, our findings support the importance of providing nutrition information/training to athletes as a strategy to support NK. This is likely to be important because higher NK is associated with more appropriate dietary intake, which can influence athletic performance. While the association between NK and diet intake has been described as ‘small’ or ‘weak’, it has also been noted that NK is an indispensable and fundamental part of achieving proper nutrition [13,38–40].

## Strengths and limitations

This study is unique in that it is the first to examine Jordanian athletes’ general and sports nutrition knowledge. Moreover, the authors also successfully translated and validated an Arabic version of the ANSKQ. Finally, we successfully recruited a very large sample size of participants; the sampling technique resulted in a sample with diverse characteristics, which allowed NK assessment in different groups to be captured; and the number of participants allowed for multivariate modelling to assess the association between these characteristics and NK.

This study does also have limitations that require mentioning. A cross-sectional design was used and therefore the results only provide a snapshot of the population at a given time, and a cause-effect relationship cannot be demonstrated. The questionnaire was administered online and thus ‘cheating’ cannot be ruled out although is unlikely given the poor NK scores. Certain demographic questions may be subject to recall and social desirability bias. A convenience sample was used; while we were able to distinguish between how long athletes had played sport and the intensity of sport played, we were not able to stratify the sample based on the level of competition played or the specific sport played, which limits ability to draw conclusions about NK of specific athletes.

## Conclusion

In conclusion, we found about 90% of Jordanian athletes and 60% of Jordanian coaches have poor general and sport nutritional knowledge. Participant’s BMI, education level, role (coach vs athlete) and attending nutrition training courses could predict NK category. Our findings suggest the need for more nutrition education among Jordanian athletes and coaches. Analyses of specific items within the ANSKQ can be used to assist researchers in developing training courses to aid in improving the general nutrition knowledge and sports nutrition knowledge level among the athletes. Future studies should further focus on: (1) the relationship between additional sports-specific factors (e.g., athletic calibre, team versus individual sports, and skills versus physique-based sports) and NK, (2) the impact of nutrition education programs in Jordanian populations, and (3) the impact of general and sports nutrition knowledge on dietary intake behaviours in Jordanian populations.

## Supporting information

S1 File Data. (XLSX)

S2 File ANSKQ questionnaire. (DOCX)
