# Supplementary material for: General and sports nutrition knowledge among Jordanian adult coaches and athletes: A cross-sectional survey
Source: PLoS One. 2021 Nov 18;16(11):e0258123. doi: 10.1371/journal.pone.0258123 (PMC8601468; doi:10.1371/journal.pone.0258123)
Supplement: S2 File — (DOCX) [file pone.0258123.s002.docx]

| **E** | |
| --- | --- |
| **General Nutrition Knowledge** |  |
| 1. Eating more energy from protein than you need can make you put on fat. (agree/disagree/not sure) |  |
| 1. The body needs fat to fight off sickness. (agree/disagree/not sure) |  |
| 1. Do you think cheddar cheese is high or low in fat? (high/low/not sure) 2. Do you think margarine is high or low in fat? (high/low/not sure) 3. Do you think honey is high or low in fat? (high/low/not sure) |  |
| 1. The body has a limited ability to use protein for muscle protein synthesis. (agree/disagree/not sure) |  |
| 1. Eggs contain all the essential amino acids needed by the body. (agree/disagree/not sure) |  |
| 1. Thiamine (Vitamin B1) is needed to take oxygen to muscles. (agree/disagree/not sure) |  |
| 1. Vitamins contain energy (kilojoules/calories). (agree/disagree/not sure) |  |
| 1. Do you think alcohol can make you put on weight? (yes/no/not sure) |  |
| 1. "Binge drinking" (also referred to as heavy episodic drinking) is generally defined as: 2. having two or more standard alcoholic drinks on the same occasion (b) having four to five or more standard alcoholic drinks on the same occasion (c) having seven to eight or more standard alcoholic drinks on the same occasion (d) Not sure |  |

| **Sport Nutrition Knowledge** |  |
| --- | --- |
| 1. Do you think 1 medium banana has enough carbohydrate for recovery from intense exercise? Assume the athlete weighs about 70 kg and has an important training session again tomorrow. (enough/not enough/not sure) 2. Do you think 1 cup of cooked quinoa and 1 tin of tuna has enough carbohydrate for recovery from intense exercise? Assume the athlete weighs about 70 kg and has an important training session again tomorrow. (enough/not enough/not sure) |  |
| 1. Do you think 100 g of chicken breast has enough protein to promote muscle growth after a bout of resistance exercise? (yes/no/not sure) 2. Do you think 1 Cup Baked Beans has enough protein to promote muscle growth after a bout of resistance exercise? (yes/no/not sure) 3. Do you think 1/2 Cup Cooked Quinoa has enough protein to promote muscle growth after a bout of resistance exercise? (yes/no/not sure) |  |
| 1. Eating more protein is the most important dietary change if you want to have more muscle. (agree/disagree/not sure) |  |
| 1. Which is a better recovery meal option for an athlete who wants to put on muscle? (a) A 'mass gainer' protein shake and 3 - 4 scrambled eggs (b) Pasta with lean beef and vegetable sauce, plus a dessert of fruit, yoghurt and nuts (c) A large piece of grilled chicken with a side salad (lettuce, cucumber, tomato)/ (d) A large steak and fried eggs (e) Not sure |  |
| 1. When we exercise at a low intensity, our body mostly uses fat as a fuel. (agree/disagree/not sure) |  |
| 1. Vegetarian athletes can meet their protein requirements without the use of protein supplement. (agree/disagree/not sure) |  |
| 1. The daily protein needs of a 100 kg (220 lb) well trained resistance athlete are closest to: 2. 100g (1g/kg) (b) 150g (1.5g/kg) (c) 500g (5g/kg) (d) They should eat as much protein as possible (e ) Not sure |  |
| 1. The optimal calcium intake for athletes aged 15 to 24 years is 500 mg. (agree/disagree/not sure) |  |
| 1. A fit person eating a balanced diet can improve their athletic performance by eating more vitamins and minerals from food. (agree/disagree/not sure) |  |
| 1. Vitamin C should always be taken by athletes. (agree/disagree/not sure) |  |
| 1. Athletes should drink water to: (a) keep plasma (blood) volume stable (b) stop dry mouth (c) allow proper sweating (d) All of the above (e) Not sure |  |
| 1. Experts think that athletes should: (a) drink 50 - 100 ml (1.7 - 3.3 fluid ounces) every 15 - 20 minutes (b) suck on ice cubes rather than drinking during practice (c) drink sports drinks (e.g. powerade) rather than water during intense sessions (d) drink to a plan, based on body weight changes during training sessions performed in a similar climate (e) Not sure |  |
| 1. Before competition, athletes should eat foods that are high in: (a) fluids, fat and carbohydrate (b) fluids, fibre and carbohydrate (c) fluids and carbohydrate (d) Not sure |  |
| 1. In events last 60 - 90 minutes, 30- 60 g (1.0 - 2.0 ounces) of carbohydrates should be consumed per hour. (agree/disagree/not sure) |  |
| 1. Eating carbohydrates when you exercise will help keep blood sugar levels stable. (agree/disagree/not sure) |  |
| 1. Which is the best snack to have during an intense 90-minute training session? (a) A protein shake (b) A ripe banana (c) 2 Boiled eggs (d) A handful of nuts (e) Not Sure |  |
| 1. How much protein do you think experts say athletes should have after completing a resistance exercise session? (a) 1.5g/kg body weight (~ 150 – 130 g/ 5.3 –10.6 ounces for most athletes) (b) 1.0 g/kg body weight (~ 50 - 100 g /1.9 - 2.3 ounces) for most athletes) (c) 0.3g/kg body weight (~ 15 - 25 g/0.53 - 0.88 ounces) for most athletes) (d) Not sure |  |
| 1. Supplement labels may sometimes say things that are not true. **(**agree/disagree/not sure) |  |
| 1. All supplements are tested to make sure they are safe and don’t have any contamination. **(**agree/disagree/not sure) |  |
| 1. Which supplement does not have enough evidence in relation to improving body composition, sporting performance? **(a)** Caffeine (b) Ferulic acid (c) Bicarbonate (d) Leucine (e) Not Sure |  |
| 1. The WORLD ANTI-DOPING AGENCY (WADA) bans the use of (a) caffeine (b) bicarbonate (c) carnitine (d) testosterone (e ) Not Sure |  |
